# The Relationship Between EFL Learners' Communication Apprehension, Self-Efficacy, and Emotional Intelligence

**DOI:** 10.3389/fpsyg.2022.847383

**Published:** 2022-02-10

**Authors:** Wenjun Cong, Pengcheng Li

**Affiliations:** ^1^School of Education, Huaibei Normal University, Huaibei, China; ^2^School of Teacher Education, Lishui University, Lishui, China

**Keywords:** communication apprehension, EFL learners, emotional intelligence, self-efficacy, emotion

## Abstract

There is ample evidence that the success or failure of language learning is influenced by psychological processes in learners' minds. This review attempted to review the related studies on the relationship between English as a Foreign Language (EFL) learners' emotional intelligence, communication apprehension, and self-efficacy. Few studies have been done on the correlation between self-efficacy and emotional intelligence. A positive significant correlation between emotional intelligence and self-efficacy has been confirmed in the literature. Studies have shown that interpersonal relationships, self-awareness, problem-solving skills, self-adequacy, self-confidence, self-actualization, and stress tolerance can act as mediator variables in the correlation between self-efficacy and emotional intelligence among EFL learners. Moreover, the related studies have shown that emotional intelligence is significantly correlated with communication apprehension. The investigations have accentuated the mediating role of learners' willingness to communicate and academic achievement in the correlation between emotional intelligence and communication apprehension. The correlation between communication apprehension and self-efficacy has been verified in the related literature. Finally, the pedagogical implications are expanded to foster language learning quality. This review also provides some suggestions for further research to elucidate our viewpoints over emotional variables and their interactions with each other.

## Introduction

Successful communication consists of the ability to transfer denotational and connotational meanings and the efficacy to employ the language knowledge on various occasions for numerous objectives. In the foreign language learning contexts, learners deal with problems in gaining this kind of proficiency and confidence in the language since they are linked to some internal mechanisms such as learning behavior, motivation, and personality types, along with external factors, including socio-economic and socio-cultural backgrounds and exposure to the language (Getie, 2020). Foreign language learners' attempts have been affected by these variables which make language learners suffer from communication apprehension when communicating in a foreign language for different purposes. Learners who do not have any confidence and efficacy to communicate efficiently in the foreign language will have to deal with the challenge of being rejected for job occasions. Learners' negative emotional constructs such as anxiety, boredom, burnout, and apprehension have been studied in more detail (Fathi and Derakhshan, 2019; Derakhshan et al., 2021a,b; Li, 2021). After the emergence of positive psychology, many positive psychologists have considered learners' strengths to improve their learning outcomes (MacIntyre et al., 2019; Wang and Huang, 2021; Wang et al., 2021; Zhang, 2021). However, this review tends to reexamine the constructs of positive affectivity in more detail with the hope to assist language learners to process language better in their minds (Bu and Kou, 2021; Du, 2021). However, this review investigates the related literature concerned with EFL learners' emotional intelligence as an umbrella term for emotional regulation, self-efficacy, and communication apprehension as a subcomponent of negative emotions in EFL contexts. However, their relationships can shed light on positive psychology constructs and their correlations with each other, and this study. Exploration of this study will be significant in the language learning education era. It has been suggested that emotional intelligence and self-efficacy may help individuals be protected against known risks of developing communication apprehension. Therefore, students should be helped to manage communication apprehension and to have successful learning. This review may help to encourage teachers and students to be more aware of students‘perceptions since according to Bandura (1993), humans' perceptions affect their actions and feelings.

## Literature Review

### The Notion of Self-Efficacy

Bandura (1986) pointed out that self-efficacy is defined as “people's judgments of their capabilities to organize and execute courses of action required to attain designated types of performances” (p. 391). He maintained that that self-efficacy highlights learners' confidence in their capability to deal with demanding tasks and to implement the required strategies to be successful in forthcoming situations. Branscombe and Baron (2016) also classified self-efficacy into self-regulatory, social self-efficacy, and academic self-efficacy. Investigations have demonstrated that learner self-efficacy can be considered a strong basis for stimulating motivation, well-being, individual achievements, and risk-taking (Fathi et al., 2020, 2021; Liang et al., 2020). Some investigations have demonstrated a significant relationship between learners' academic achievement and self-efficacy (e.g., Qusay, 2020; Bouih et al., 2021; Özer and Işpinar Akçayoglu, 2021; Zheng et al., 2021a,b). Therefore, if a learner has a high level of self-efficacy, his accomplishment in educational contexts should be remarkable. Moreover, some investigations have revealed a significant correlation between EFL learners' self-efficacy and language skills. These studies emphasized the role of promoting learners' self-efficacy to have pleasant results in language contexts (McLean and Poulshock, 2018; Chen and Zhang, 2019; Haerazi and Irawan, 2020; Chung et al., 2021).

### The Notion of Emotional Intelligence

Fernández-Abascal and Martín-Díaz (2015) defined emotional intelligence as one's capability to decide on his/her judgments, emotional states, and activities craftily and deliberately, so as to achieve optimal consequences. Zeidner et al. (2002) argued that emotional intelligence, a subcategory of social intelligence, includes assessment, manifestation, use, and managing one's own affections and feelings of others. Vesely et al. (2013) argued that the growth of capabilities is affected by emotional intelligence which develops emotional health. Allen et al. (2014) stated that emotional intelligence should be regarded as an important element for developing character strengths. In a foreign language learning context, MacIntyre et al. (2019) asserted that teaching emotional intelligence and positive psychology constructs can develop “learners' interpersonal relationships, positive emotions, greater well-being, and so on” (p. 5). Moreover, Oxford (2016) highlights the role of language instructors in helping learners improve and use their emotional intelligence in EFL contexts to have interpersonal and intrapersonal relationships with peers and teachers. Also, there are some investigations that verify a significant relationship between EFL learners' academic achievements and their emotional intelligence in instructive contexts (e.g., Lea et al., 2018; Halimi et al., 2021).

### The Notion of Apprehension

Kyriacou (2001) defined apprehension as the future–based anxiety and stress which occur in unpleasant specialized contexts. According to Gardner and MacIntyre (1993) “anxiety is fear or apprehension occurring when a learner is expected to perform in the second or foreign language” (p. 59). Investigations in the realm of communication have emphasized the role of communication comprehension in foreign language contexts (Hasni et al., 2019). According to Horwitz et al. (1986) communication apprehension is “a distinct complex of self-perceptions, beliefs, feelings, and behavior related to classroom language learning arising from the uniqueness of the language-learning process” (p. 28). Mahdi's (2015) study revealed a significant relationship between EFL learners' communication apprehension and communicative competence. He argued that learners' anxiety is regarded as a barrier in interaction and language learning. Instead, he offered supplementary activities which help learners to have chances to communicate through the target language. Communication apprehension has a significant correlation with language learners' linguistic background and their proficiency levels (Molnar and Crnjak, 2018; Botes et al., 2020). Spetz (2018) investigated Swedish foreign language learner communication apprehension, and he attributed it to the level of proficiency in language learners as beginners have a higher level of communication apprehension. Shan et al. (2020) attributed foreign language communication apprehension to numerous factors such as deficient tasks in foreign language learning contexts, lack of input flood, first language overuse, and numerous linguistic, emotional, and socio-cultural issues.

### The Relationship Between EFL Learners' Self-Efficacy and Emotional Intelligence

Some investigations have been done on the correlation between self-efficacy and emotional intelligence, particularly among instructors in educational contexts. Kostić-Bobanović (2020) found out that teachers' self-efficacy is significantly correlated with their emotional intelligence. Their study revealed that teachers' interpersonal relationships, affective self-awareness, and problem-solving skills are significantly correlated with their self-efficacy. Fathi et al. (2021) found a significant bidirectional relationship between EFL teachers' emotion regulation, as a subcomponent of emotional intelligence, and self-efficacy. They argued that knowledgeable instructors who can control their classrooms, employ instructional methodologies, and encourage learners to do tasks are competent in emotion regulation while working their professional affairs. Regarding the correlation between EFL learners' self-efficacy and emotional intelligence, there have been few studies that highlighted this issue. Adeyemo (2007) found a moderating role of EFL learners' emotional intelligence in the correlation between their academic self-efficacy and academic success. They argued that learners with higher levels of emotional intelligence have higher levels of self-adequacy, self-confidence, and self-efficacy. In another study, Hashemi and Ghanizadeh (2011) revealed that EFL learners' emotional intelligence is significantly associated with self-efficacy beliefs. They stated that stress forbearance and self-actualization are significantly correlated with EFL learners' self-efficacy. They argued that foreign language learners' confidence in their capability to be successful in language learning is affected by their emotional intelligence. They claimed that learners' feelings can influence their self-efficacy. Abdul Rashid et al. (2020) argued that learners' academic self-efficacy is significantly correlated with their emotional intelligence. They maintained that learners' emotional intelligence depends on their self-efficacy. Therefore, self-efficacy influences emotional intelligence individually and socially, and it can expedite learners' learning experience.

### The Relationship Between EFL Learners' Emotional Intelligence and Communication Apprehension

Since emotional intelligence relates to learners' feelings and emotional states, investigators have examined its association with other affective variables. Shao et al. (2013) revealed that communication apprehension plays a moderator role in the correlation between EFL learners' emotional intelligence and learners' English proficiency. They argued that EFL learners who are emotionally intelligent control their affective reactions, control stress, and can be self-confident over time. Therefore, they have the effective capability to talk with peers and instructors in a foreign language without any apprehension and anxiety. They also argued that the emotional intelligence construct includes all possible emotional states and foreign language apprehension is a type of emotion in FLA. It is reasonable to presume that learners with higher levels of emotional intelligence have low-level apprehension in foreign language communication. Mehrpoor and Soleimani (2018) examined the correlation between EFL learners' communication apprehension, emotional intelligence, and willingness to communicate. Their study revealed that emotional intelligence is regarded as an external stimulus for interpersonal communication. They found out that learners' emotional intelligence is significantly correlated with their willingness to communicate. However, their study demonstrated that EFL learners' emotional intelligence has a negative correlation with their communication apprehension. They argued that EFL learners with high levels of emotional intelligence can control their negative emotions under adverse states and efficiently start communication in L2. Moreover, they can overcome communication apprehension. Therefore, it can be argued that willingness to communicate acts as a moderator variable in the negative correlation between EFL learners' emotional intelligence and communication apprehension. Ateş's (2019) study revealed that EFL learners' emotional intelligence significantly predicted their reading comprehension and reading apprehension as a subcomponent of communication apprehension. Huerta et al. (2016) verified a significant relationship between ESL learners' self-efficacy and their writing apprehension. However, they did not find a direct correlation between ESL learners' writing apprehension and their emotional intelligence. They argued that ESL learners' foreign language apprehension and emotional intelligence are related to their performance and achievements in EFL contexts.

### The Relationship Between EFL Learners' Communication Apprehension and Self-Efficacy

Some investigations have demonstrated that communication apprehension has a significant relationship with some definite emotional states which are significantly related to EFL learners' proficiency. Self-efficacy is one of these factors which contributes to diminishing learners' communication apprehension (Tsai, 2013). Mede and Krairmak (2017) found out learners' communication apprehension is strongly correlated with their self-efficacy. They argued that learners with a low level of self-efficacy cannot reach their objectives, and therefore they feel nervous and depressed during communication. Tuncer and Dogan (2016) also found out that self-efficacy negatively influences communication apprehension. They argued that learners with low levels of self-efficacy can reflect that tasks are more challenging than they actually are, and this increases their anxiety in foreign language contexts. They argued that apprehended learners with low levels of self-efficacy act poorly in academic contexts. Tahsildar and Kabiri (2019) found out that anxious and self-efficient EFL learners do not focus on their speaking skill. Shirkhani and Mir Mohammad Meigouni (2020) investigated the correlation between EFL learners' communication strategy use, their self-efficacy, and communication apprehension. They found out that learners who use communication strategies are likely to enhance their self-efficacy and decrease their communication apprehension. They argued that learners, who choose better communication strategies, are usually self-efficient and communicatively competent and this results in achievement in foreign language learning.

## Implications and Suggestions

This paper reviewed the related literature on the relationship between EFL learners' communication apprehension, self-efficacy, and emotional intelligence. Learners can develop their emotional intelligence through increasing self-efficacy and decreasing their apprehension during their communication and giving presentations. Thus, this review implicates that learners should be conscious about regulating, modifying, managing their emotional intelligence, and anxiety in foreign language environments. In addition to self-efficacy and emotional intelligence, learners are required to have communication competence to be successful in language learning. Language instructors are required to use a proactive approach and they should try to provide pleasant contexts for learners through integrating enjoyment in class in order to raise learners' emotional intelligence and self-efficacy and they should reduce stress sources among learners. They can boost EFL learners' self-efficacy by giving confidence in EFL classrooms. Some helpful instructional materials should be selected to increase learners' emotional intelligence and self-efficacy and to lessen negative emotions such as communication apprehension among EFL learners. The instructors are required to enhance stimulating and pleasant language learning tasks which arouse their emotional intelligence and to lessen communication apprehension in their minds. Consequently, the presentation of pleasurable tasks can help learners manage their learning emotional intelligence, self-efficacy, and language anxiety to improve their communicative competence. These issues diminish learners' cognitive load and develop their academic achievement in foreign language contexts (Piniel and Albert, 2018). Besides, EFL instructors should be aware of learners' personalities, and they should identify learners' requirements and guide them to explore their own solutions for the problems in language learning contexts. Teacher educators and mentors should offer some techniques for instructors to advance learners' emotional intelligence, self-efficacy, and to decrease their communication apprehension. They should help teachers to provide some classroom tasks including problem-solving strategies, and they should encourage teachers to organize group activities. Workshops about positive and negative emotions can be organized by teacher educators, school principals, and policy-makers for pre-service and in-service teachers to help them cope with the problems of learners' communication apprehension and increase their self-efficacy in foreign language learning.

This review has some suggestions for further research. Future studies can validate numerous measures of learners' emotional intelligence. Investigations need to be done to study learners' emotional intelligence and their anxiety in communication in numerous instructive, local, national, and cultural contexts. Some studies should be done on learners' mindset and its relation with their communication apprehension, self-efficacy, and emotional intelligence. Regarding emotional intelligence, the effect of instructors' methodologies on learners' emotional intelligence should be investigated. Besides, future studies can focus on gender effects on foreign language learners' emotional intelligence. Moreover, investigations are required to explore the influence of video games on learners' emotional intelligence. In addition, future studies can examine the effects of emotional psychology on EFL learners' working memory. Also, the effects of EFL learners' emotional intelligence on the development of language skills should be considered in detail. Language teaching approaches have been affected by online language learning during the COVID-19 pandemic. Future research should enlighten the role of the COVID-19 pandemic on learners' self-efficacy and emotional intelligence in traditional and virtual contexts to illuminate the influence of learning contexts on learners' emotional traits. The effect of third language knowledge and pedagogical background on learners' emotional intelligence is required to be investigated. Regarding self-efficacy and communication apprehension, further studies need to be done to elucidate the impact of teacher burnout on learners' communication self-efficacy and apprehension. Moreover, the relationships between learners' positive psychology constructs and negative emotional components such as communication apprehension are worth to be studied. Resilience and its relationship with self-efficacy should be inspected in more detail.

## Author Contributions

WC took responsibility for the design and implementation of the research. PL wrote the review. All authors contributed to the article and approved the submitted version.

## Funding

The research project is the general project of Anhui provincial philosophy and social science planning-research achievement in Research on formation mechanism and cultivation path of disabled undergraduates' positive social mentality (No.: AHSKY2018D33).

## Conflict of Interest

The authors declare that the research was conducted in the absence of any commercial or financial relationships that could be construed as a potential conflict of interest.

## Publisher's Note

All claims expressed in this article are solely those of the authors and do not necessarily represent those of their affiliated organizations, or those of the publisher, the editors and the reviewers. Any product that may be evaluated in this article, or claim that may be made by its manufacturer, is not guaranteed or endorsed by the publisher.
